# The Enhancement of Intestinal Immunity in Offspring Piglets by Maternal Probiotic or Synbiotic Supplementation Is Associated With the Alteration of Gut Microbiota

**DOI:** 10.3389/fnut.2021.686053

**Published:** 2021-07-09

**Authors:** Kai Wang, Chengjun Hu, Wu Tang, Md. Abul Kalam Azad, Qian Zhu, Qinghua He, Xiangfeng Kong

**Affiliations:** ^1^Key Laboratory of Agro-ecological Processes in Subtropical Regions, Hunan Provincial Key Laboratory of Animal Nutritional Physiology and Metabolic Process, National Engineering Laboratory for Pollution Control and Waste Utilization in Livestock and Poultry Production, Institute of Subtropical Agriculture, Chinese Academy of Sciences, Changsha, China; ^2^Department of Food Science and Engineering, College of Chemistry and Environmental Engineering, Shenzhen University, Shenzhen, China

**Keywords:** Bama mini-pigs, immune function, intestinal microbiota, probiotic, synbiotic

## Abstract

A total of 64 pregnant Bama mini-pigs were used to investigate the effects of maternal probiotic or synbiotic supplementation during gestation and lactation on immune response, intestinal morphology, and microbiota community of offspring piglets. The sows were assigned randomly to one of four groups, control group (basal diet), antibiotic group (basal diet supplemented with 50 g/t virginiamycin), probiotic group (basal diet supplemented with 200 mL/d probiotic fermentation broth per pig), or synbiotic group (basal diet supplemented with 200 mL/d probiotic fermentation broth per pig + 500 g/t xylo-oligosaccharides) during pregnancy and lactation periods. After weaning, two piglets close to the average body weight (BW) per litter were selected and fed a basal diet. Eight piglets with similar BW were selected from each group for sample collection at 65 d-old. The results showed that plasma interleukin (IL)-2 and lipopolysaccharide concentrations were decreased (*P* < 0.05) in the probiotic group, while the immunoglobulin A (IgA) concentration in the probiotic and synbiotic groups was increased (*P* < 0.05), when compared with the control group. The jejunal IL-10, interferon-α, and secretory IgA (sIgA) concentrations were increased (*P* < 0.05) in the probiotic and synbiotic groups, as well as the ileal sIgA concentration in the probiotic group. The jejunal villus height (VH) and the ratio of VH to crypt depth were increased (*P* < 0.05) in the probiotic group, as well as the ileal VH in the synbiotic group. Furthermore, the piglets from the antibiotic group exhibited a lower microbiota diversity in the jejunum and ileum. The piglets from the synbiotic group had higher relative abundances of *Actinobacteria, Bifidobacterium, Turicibacter*, and *Clostridium* in the jejunum compared with the antibiotic group. Dietary probiotic treatment increased (*P* < 0.05) the relative abundance of *Psychrobacter* in the ileum compared with the antibiotic and control groups. Spearman's correlation analysis revealed that the relative abundances of *Bifidobacterium, Clostridium*, and *Blautia* in the jejunum and *Psychrobacter* in the ileum, were positively correlated with the alterations of immunoglobulin and cytokines. Collectively, these findings suggest that maternal interventions with probiotic or synbiotic are promising strategies for improving the immune response of offspring piglets by altering the gut microbiota.

## Introduction

The gut microbiota exerts important roles in gut function, host metabolism, immune development, and cell proliferation and differentiation during early life (1, 2). Early colonization of intestinal microbiota promotes the development and maturation of the intestinal immune system and barrier function of the host (3). Maternal diet during pregnancy and lactation periods is one of the pivotal factors affecting the growth and development of the offspring (4). During pregnancy and lactation, maternal body undergoes substantial hormonal, immunological, and metabolic changes that affect their gut microbiota (5). Although still controversial, more and more evidence suggests that the mother's intestine microbes could colonize the fetus *in utero* (6–8). Therefore, maternal microbiota could be transmitted to the offspring through direct contact during parturition or breastfeeding during lactation (9, 10). In humans, it has also been proposed that the administration of probiotics during the perinatal and lactation periods favors beneficial bacteria colonization in the infant's gut (11, 12). Recent studies also showed that maternal commensal microbiota can induce antibodies that recognize antigens expressed by pathogenic bacteria and protect offspring from infection (13). In this context, increasing the maternal beneficial microbiota through dietary supplementation during gestation and lactation periods has been considered a window of opportunity to modulate the intestinal health in offspring.

A previous study has reported that supplementing *Enterococcus faecium* to sows' diets during late pregnancy and lactation periods improved the offspring's body weight and average daily gain, and altered the fecal microbiota (14). Supplementing *Bacillus subtilis* to sows' diets during gestation and lactation periods reduced pathogenic bacteria and established beneficial bacteria (such as *Lactobacillus*) in neonatal piglets (15). Our previous studies showed that dietary probiotics supplementation to sows improved the birth weight of suckling piglets (16). Moreover, dietary synbiotics supplementation improved the piglet survival rate, but did not affect the litter size, born alive, and average body weight (17). In addition, maternal synbiotic supplementation increased the average daily gain of 65 d-old piglets; and maternal probiotic or synbiotic supplementation reduced the feed/gain ratio of 65 d-old piglets (unpublished data). However, the effects of supplementing probiotic or synbiotic to sows' diets on the immune system and gut microbiota composition of weaned piglets are poorly understood. Therefore, the present study aimed to determine the effect of maternal probiotic or synbiotic supplementation on the gut microbiota and immune response in offspring weaned piglets.

## Materials and Methods

### Animals, Treatments, and Diets

A total of 64 pregnant Bama mini-pigs during 3–5 parities with similar initial body weight (about 90 kg) were randomly assigned to one of four groups, with 16 sows per group. The four groups were as follows: control group (basal diet), antibiotic group (basal diet supplemented with 50 g/t virginiamycin), probiotic group (basal diet supplemented with probiotic fermentation broth 200 mL/d per pig), and synbiotic group [basal diet supplemented with probiotic fermentation broth 200 mL/d per pig + 500 g/t xylo-oligosaccharides (XOS)]. The compound probiotic fermentation broth was purchased from Hunan LiFeng Biotechnology Co., Ltd. (Changsha, China) and contained ≥1.2 × 10^8^ CFU/g viable bacteria (*Lactobacillus Plantarum* B90 CGMCC1.12934 ≥1.0 × 10^8^ CFU/g and *Saccharomyces cerevisiae* P11 CGMCC2.3854 ≥0.2 × 10^8^ CFU/g). The viable microbe number in the broth was counted by the plate counting method. Then the fermentation broth was mixed with the diet according to the dose mentioned above per day before feeding. The XOS was purchased from Shandong Longlive Biotechnology Co., Ltd. (Shandong, China) and composed of xylobiose, xylotriose, and xylotetraose (accounting for ≥35%), which met the Chinese feed additive recommended requirements of XOS (GB/T23747-2009). The dose of compound probiotic and XOS is referred to the previous studies (17, 18).

After insemination, the sows were individually housed in gestation crates (2.2 m × 0.6 m) from day 0 (mating) to day 105 of pregnancy and then moved to the individual farrowing crates (2.2 m × 1.8 m) until weaning. The sows were fed 0.8, 1.0, 1.2, 1.5, and 2.0 kg of pregnant diets during 1–15, 16–30, 31–75, 76–90, and 91–105 days of pregnancy, respectively, fed 1.0 kg pregnant diets before a week of parturition, and *ad libitum* from delivery to weaning. The sows were allowed to drink freely throughout the trial (19).

The piglets were weaned at 28 ± 3 days and offered creep feed for 1 week. After 1 week of adaption, two piglets close to the average body weight (BW) per litter were transferred to the nursery facility and paired with two piglets from another sow within the same treatment. Four piglets were assigned to one pen, with eight pens (replicates) per group. The piglets were allowed *ad libitum* consumption of basal diets (provided twice daily; at 8:00 and 17:00). Each pen was equipped with a single-hole feeder and a water nipple to allow *ad libitum* access to water for sows and piglets. The composition and nutrients levels of basal diets for sows and piglets met the Chinese nutrient requirements of swine in China (NY/T65-2004), and the premixes met the NRC recommended requirements (20) (Supplementary Tables 1, 2).

### Sample Collection

Eight piglets (one piglet per pen) were randomly chosen from each group, fasted for 12 h, and then weighed at 65 d-old. Blood samples were collected *via* the precaval vein of each pig and centrifuged at 3,500 × g for 10 min at 4°C to obtain the plasma and then stored at −20°C for future analyses. Following the blood sampling, all piglets were exsanguinated after electrical stunning (120 V, 200 HZ). The contents of the jejunum (10 cm below the flexure of duodenum-jejunum) and ileum (10 cm above the ileocecal junction) were collected and stored at −80°C for microbial composition and metabolites analyses. The jejunum and ileum segments (~2 cm in length) were washed with phosphate buffered saline (PBS) to remove intestinal contents and then fixed with 4% paraformaldehyde for analyzing intestinal morphology. The jejunum and ileum tissues were excised and washed with PBS. Then the mucosa samples were collected and immediately stored at −80°C for cytokines and secretory immunoglobulin A (sIgA) analyses.

### Analysis of Plasma Cytokines, Immunoglobulins, Endotoxin, and D-Lactic Acid

The plasma concentrations of interleukin-2 (IL-2), IL-6, IL-10, tumor necrosis factor-alpha (TNF-α), interferon-alpha (IFN-α), interferon-gamma (IFN-γ), IgA, IgM, lipopolysaccharide (LPS), and D-lactic acid (D-LA) were determined using ELISA assay kits (Mei mian, Jiangsu, China) according to the manufacturer's protocol.

### Analysis of Intestinal Cytokines and sIgA Levels

The jejunal and ileal mucosa samples about 0.1000 g were homogenized with ice-cold physiological saline (1:9, w/v) and centrifuged at 2,000 × *g* for 20 min at 4°C, and then the supernatants were separated for determining cytokines and sIgA levels. The levels of IL-2, IL-6, IL-10, TNF-α, IFN-α, IFN-γ, and sIgA in the intestinal mucosa were measured using ELISA assay kits (Mei mian, Jiangsu, China) according to the manufacturer's protocol. The total protein levels of the supernatants were quantified using the Pierce BCA Protein Assay Kit (Thermo Scientific, Shanghai, China). The intestinal cytokines and sIgA levels were normalized to the total protein concentration (mg/L) of the supernatants.

### Intestinal Histomorphology Analysis

The jejunum and ileum segments fixed with 4% paraformaldehyde solution were dehydrated and then embedded in paraffin. The tissues were cut into ~5-μm thick sections. Hematoxylin and eosin (H&E) staining was performed according to the previously described protocol (21). The digital images of intestinal morphology at 40 and 100 × magnification were captured using a light microscope (Olympus Bx51, Japan). Fifteen representative measures per tissue were used to measure the villus height (VH) and crypt depth (CD) using Image-Pro Plus software (version 6.0) in a blinded manner. The VH was defined as the distance from the crypt-villus junction to the tip of the villus, and the CD was defined as the distance from the bottom of the crypt to the crypt-villus junction, as described previously (22).

### Microbial Metabolite Analysis

The short-chain fatty acids (SCFAs), including acetate, propionate, butyrate, isobutyrate, valerate, and isovalerate in the jejunum and ileum contents were determined by gas chromatography (Agilent Technologies 1206, Santa Clara, CA), as reported previously (23).

### Intestinal Microbiota Analysis

Total microbial genomic DNA was extracted from the jejunal and ileal contents (~0.3000 g) using Mag-Bind® Stool DNA Kit (Omega, Guangzhou, China) following the manufacturer's instructions and stored at −20°C for further analysis. The concentration and quality of the extracted DNA were confirmed using a NanoDrop OneC Microvolume UV-Vis Spectrophotometer (Thermo Scientific, Waltham, USA) and 1.2% agarose gel electrophoresis. The V3–V4 region of the 16S rRNA genes were amplified with primers: 338F (5′-ACTCCTACGGGAGGCAGCA-3′) and 806R (5′-GGACTACHVGGGTWTCTAAT-3′). The PCR amplicons were purified with Vazyme VAHTSTM DNA Clean Beads and quantified using the Quant-iT PicoGreen dsDNA assay kit (Invitrogen, Carlsbad, USA). Purified amplicons were sequenced on an Illumina MiSeq platform (Illumina, San Diego, USA) with the MiSeq Reagent Kit v3 (600 cycles) according to the standard protocol by Shanghai Personal Biotechnology Co. Ltd. (Shanghai, China).

The QIIME2 pipeline was used to process the sequencing data as previously described (24). Briefly, raw sequencing reads exactly matched with the barcodes were assigned to each sample and identified as valid sequences. Low quality sequences were filtered by the following criteria (25): sequences lengths shorter than 150 bp, an average Phred score lower than 20, and ambiguous bases or mononucleotide repeats exceeding 8 bp. Paired-end reading was calculated using FLASH (v1.2.11) assembly (26). After chimera detection, the remaining high-quality sequences were clustered into operational taxonomic unit (OTU) with 97% sequence identity using USEARCH (v7.0.1090) (27). A representative sequence was selected from each OTU using default parameters. The OTU taxonomic classification was conducted by Ribosomal Database Project (RDP) Classifier trained on Greengene (V201305) reference database (28). All the raw Illumina pair-end read data involved in the present study were deposited in the NCBI Sequence read archive (SRA) database under accession number PRJNA718786.

### Statistical Analyses

The plasma and intestinal immune parameters were analyzed by one-way ANOVA and the comparative analysis was conducted using the Tukey *post-hoc* test (SPSS 22.0; SPSS Inc., Chicago, IL, USA). The microbiota alpha diversity and abundance were analyzed by the Kruskal-Wallis rank-sum test. All data are presented as means ± SEM. The significance value and a trend toward difference were set at levels of *P* < 0.05 and 0.05 ≤ *P* < 0.10, respectively. Alpha diversity (including Chao1, Shannon, Simpson, and Observed_species) was assessed using the OTU table in QIIME2 software. Partial least squares discriminant analysis (PLS-DA) based on unweighted UniFrac distance with constrained ordination and supervised learning was performed to reveal the intestinal microbiota variation among samples. The relative abundances of jejunal and ileal microbiota at phylum and genus levels were analyzed by Meta stats (http://metastats.cbcb.umd.edu/). Linear discriminant analysis (LDA) effect size (LEfSe) analysis was used to compare differences at the genus level among the four groups. The Spearman correlations between immune-related indexes and intestinal microbiota were measured using the R package ggplot2 3.3.1 (https://www.r-project.org/).

## Results

### The Concentrations of Plasma Cytokines and Immunoglobulins in Offspring Piglets

**Figure 1:**
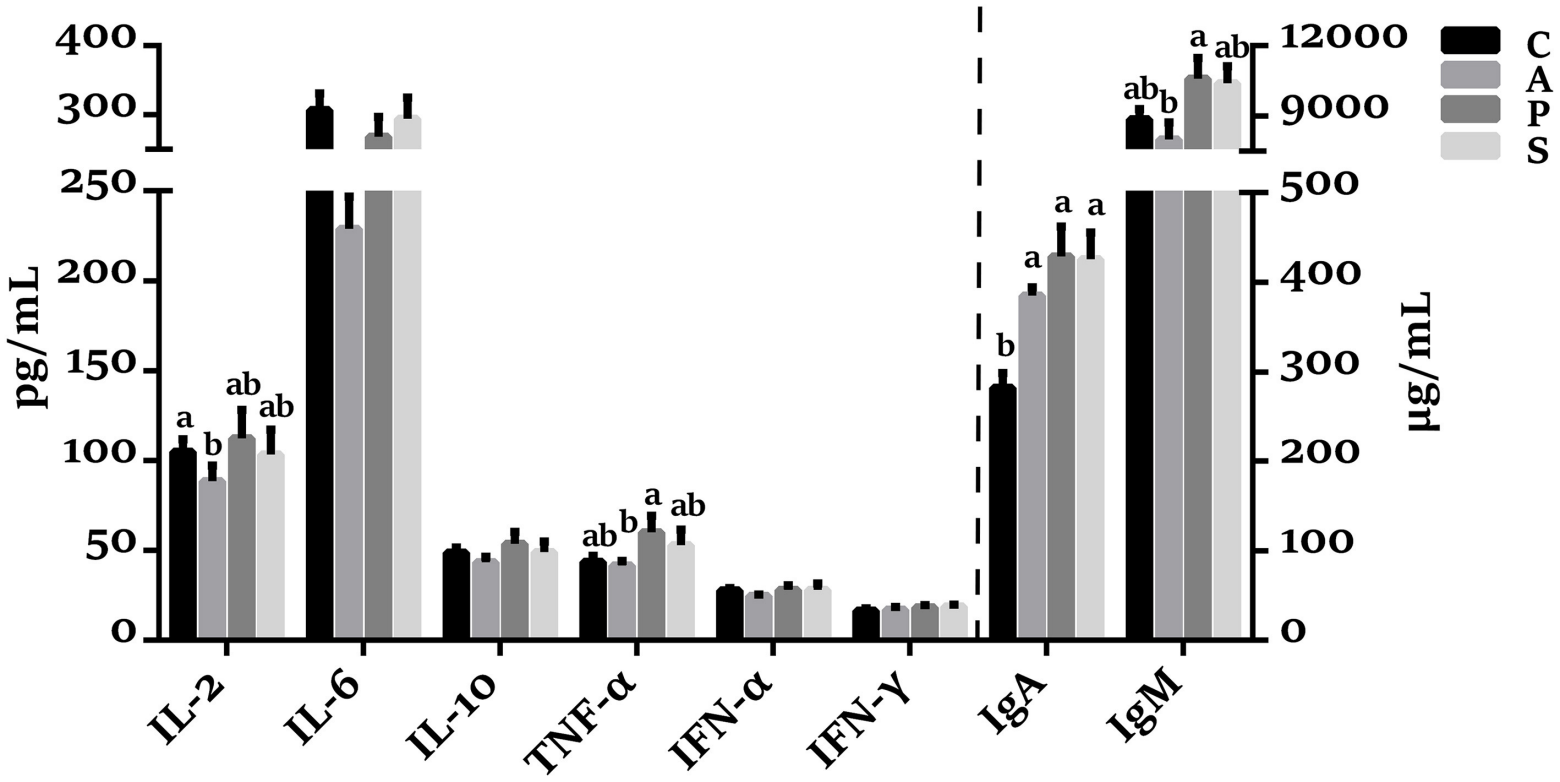
Effects of maternal probiotic or synbiotic supplementation on the plasma cytokines concentrations of offspring piglets. Data are represented as means ± SEM, *n* = 8 per group. Different letters indicate significant differences among the groups (*P* < 0.05). IL, Interleukin; TNF-α, Tumor necrosis factor-alpha; IFN, Interferon; IgA, immunoglobulin A; IgM, immunoglobulin M; C, control group; A, antibiotic group; P, probiotic group; and S, synbiotic group.

As shown in Figure 1, the plasma concentrations of TNF-α and IgM in the probiotic group were higher (*P* < 0.05) than in the antibiotic group. The IgA concentration in the antibiotic, probiotic, and synbiotic groups was increased (*P* < 0.05) compared with the control group.

### The Concentrations of Cytokines and sIgA in the Jejunum and Ileum of Offspring Piglets

**Figure 2:**
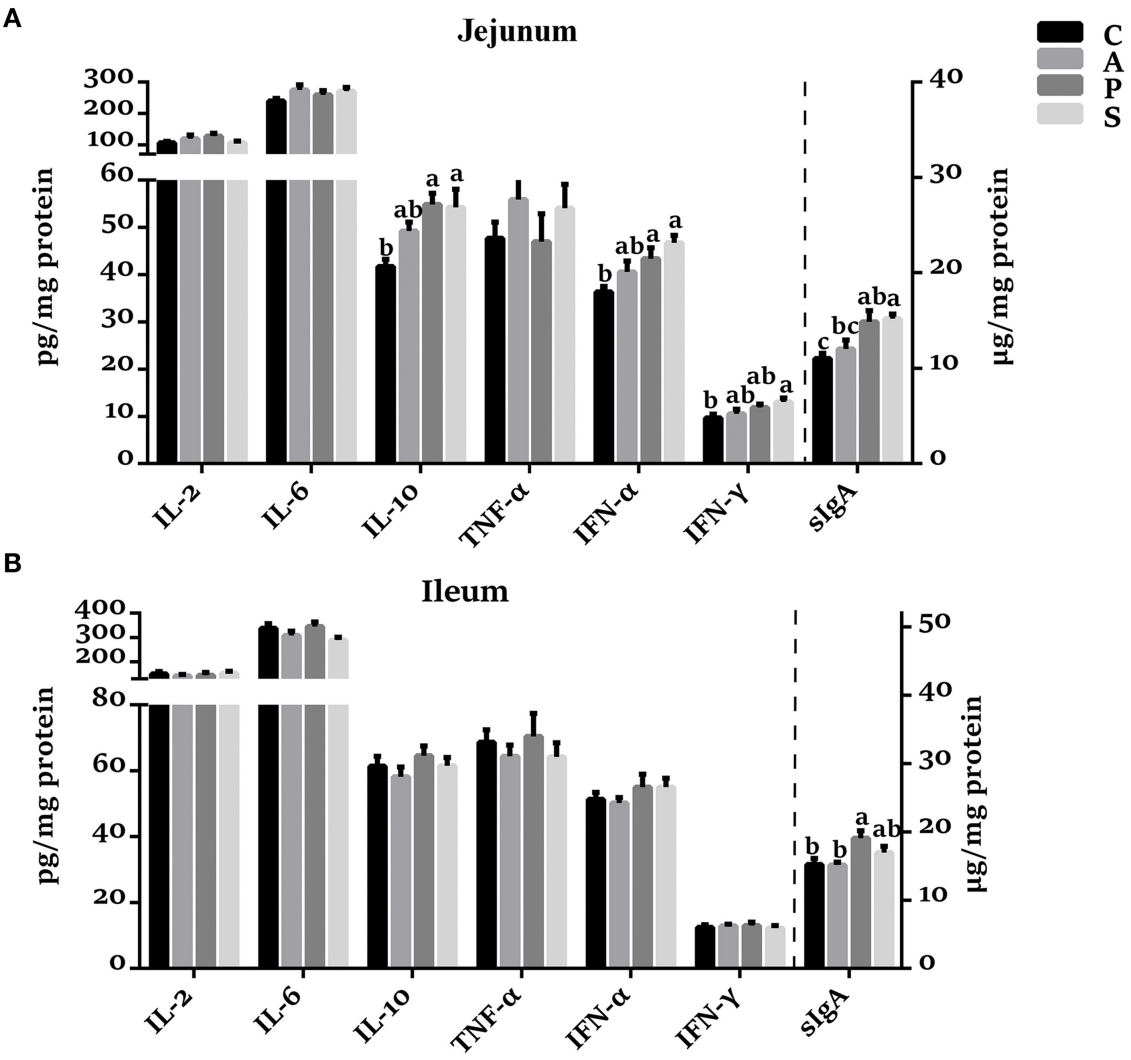
Effects of maternal probiotic or synbiotic supplementation on the concentrations of cytokines and sIgA in the **(A)** jejunum and **(B)** ileum of offspring piglets. Data are represented as means ± SEM, *n* = 8 per group. Different letters indicate significant differences among the groups (*P* < 0.05). IL, Interleukin; TNF-α, Tumor necrosis factor-alpha; IFN, Interferon; sIgA, secretory immunoglobulin A; C, control group; A, antibiotic group; P, probiotic group; and S, synbiotic group.

In the jejunal mucosa, the concentrations of IL-10, IFN-α, and sIgA were increased (*P* < 0.05) in the probiotic and synbiotic groups, as well as IFN-γ in the synbiotic group, when compared with the control group (Figure 2A). The sIgA concentration in the synbiotic group was higher (*P* < 0.05) than in the antibiotic group. However, no significant differences were observed in the concentrations of IL-2, IL-6, and TNF-α among the four groups. In the ileal mucosa, probiotic treatment increased (*P* < 0.05) the sIgA concentration compared with the control and antibiotic groups (Figure 2B).

### The Morphology and Permeability in the Jejunum and Ileum of Offspring Piglets

**Figure 3:**
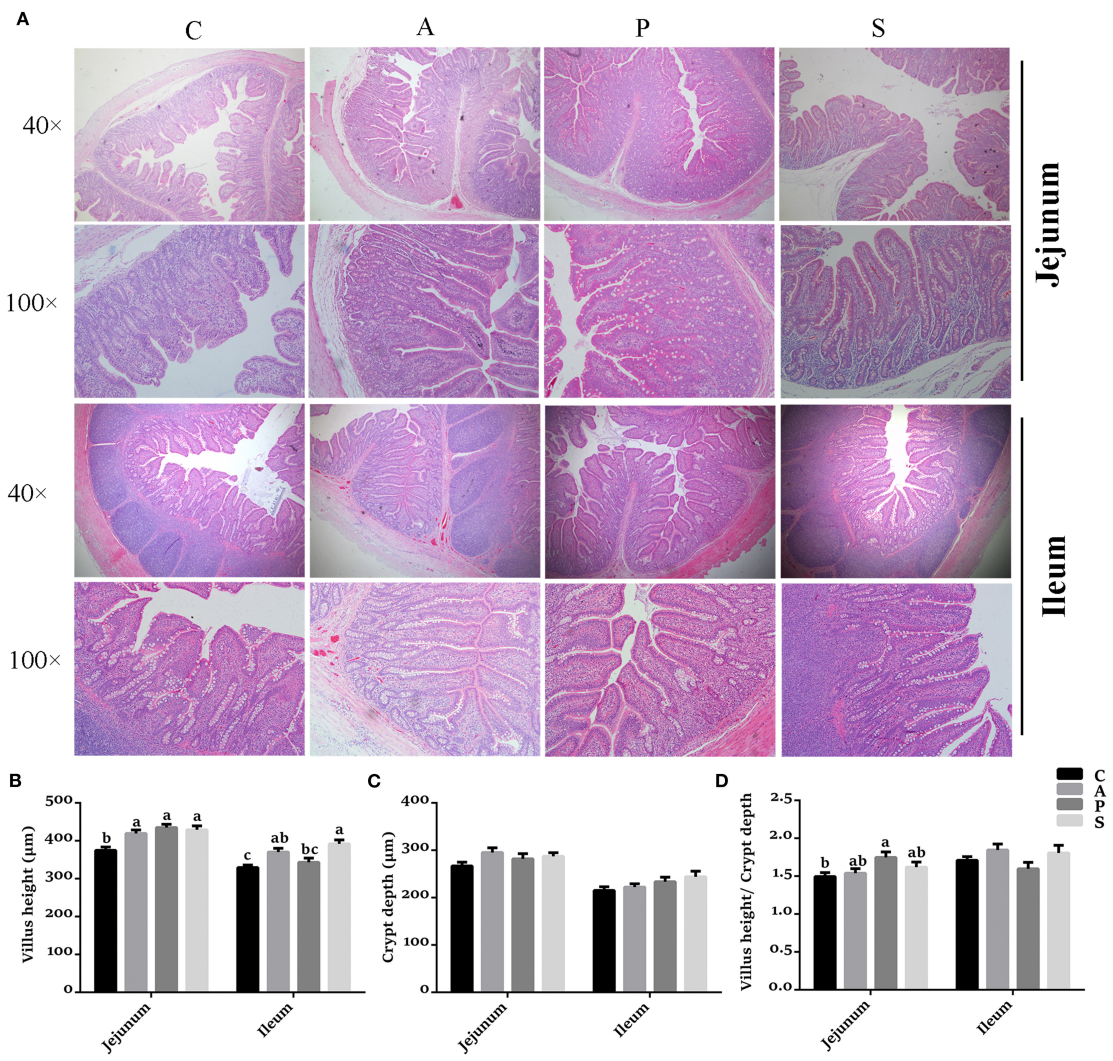
Effects of maternal probiotic or synbiotic supplementation on the intestinal morphology of offspring piglets. Histomorphology of the jejunum and ileum was observed by light microscopy after H&E staining. **(A)** The images of the jejunum and ileum morphology at 40 × and 100 × magnification were shown, respectively. **(B)** Statistical analysis of villus height (VH), **(C)** crypt depth (CD), and **(D)** the ratio of VH to CD. Data are represented as means ± SEM, *n* = 8 per group. Different letters indicate significant differences among the groups (*P* < 0.05). C, control group; A, antibiotic group; P, probiotic group; and S, synbiotic group.

**Figure 4:**
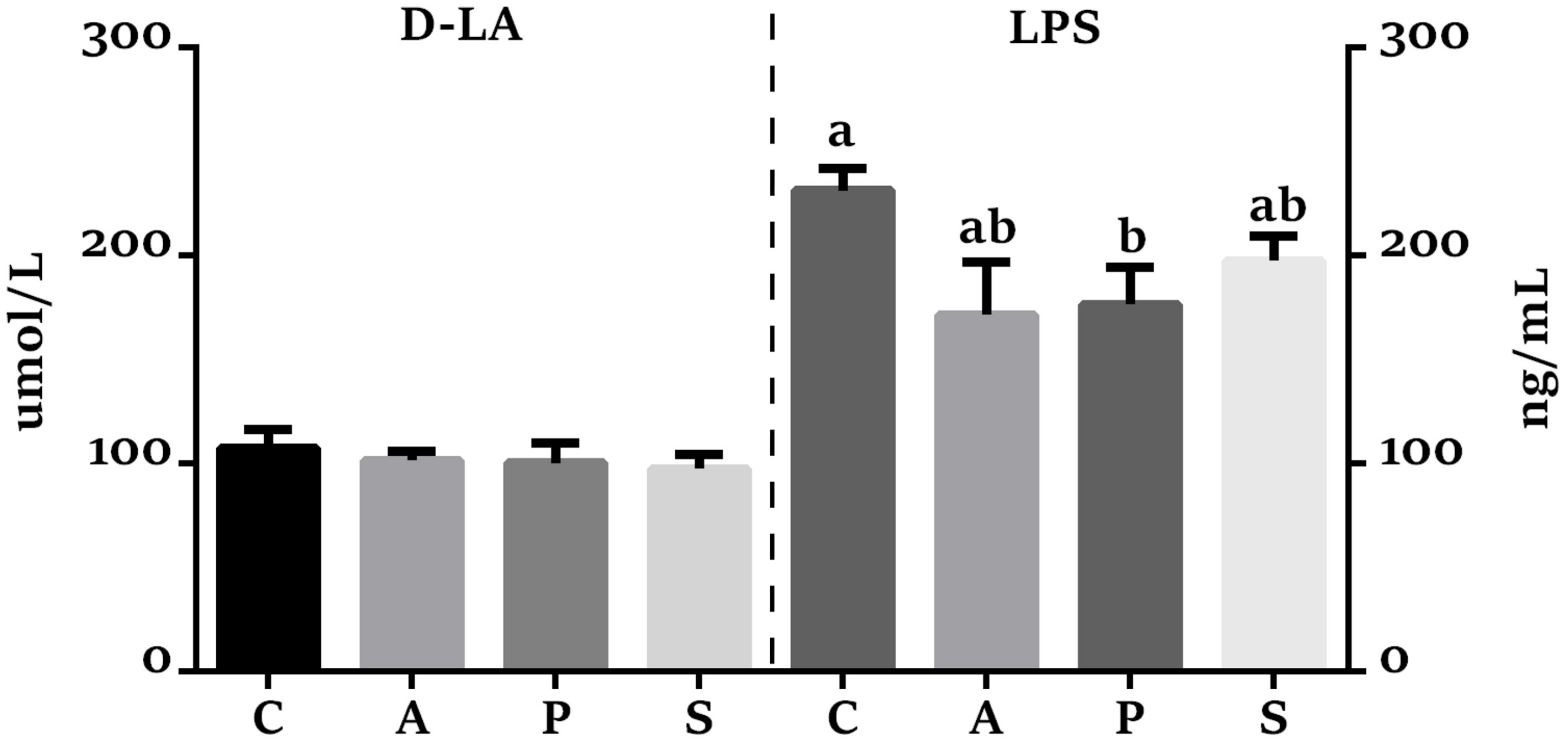
Effects of maternal probiotic and synbiotic supplementation on the plasma concentrations of D-lactate (D-LA) and Lipopolysaccharide (LPS) of offspring piglets. Data are represented as means ± SEM, *n* = 8 per group. Different letters indicate significant differences among the groups (*P* < 0.05). C, control group; A, antibiotic group; P, probiotic group; and S, synbiotic group.

Compared with the control group, the VH in the jejunum was higher (*P* < 0.05) in the antibiotic, probiotic, and synbiotic groups (Figures 3A,B), while the ratio of VH to CD was higher (*P* < 0.05) in the probiotic group (Figures 3A,D). The VH in the ileum was higher (*P* < 0.05) in the antibiotic and synbiotic groups compared with the control group. However, no significant difference were observed in the CD of jejunum and ileum (Figure 3C). In addition, the plasma LPS level in the probiotic group was decreased (*P* < 0.05) compared with the control group (Figure 4).

### The SCFAs Concentrations in the Jejunum and Ileum of Offspring Piglets

The ileal acetate concentration was higher by 50.8% in the synbiotic group than in the control group, but there was no statistically significant difference (Supplementary Table 3). In addition, the concentrations of propionate, valerate, isobutyrate, and isovalerate in several samples were below the detection level.

### The Microbiota Diversity in the Jejunum and Ileum of Offspring Piglets

**Figure 5:**
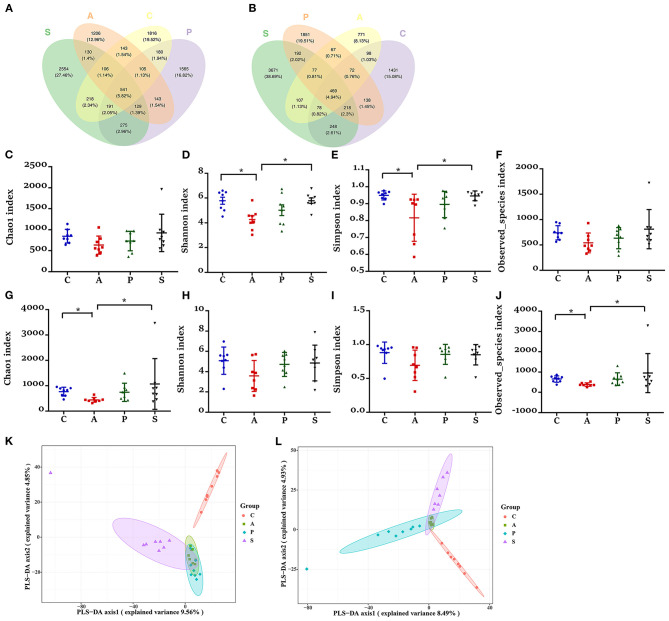
Effects of maternal probiotic and synbiotic supplementation on microbial alpha diversity in the jejunum and ileum of offspring piglets. Venn diagrams for microbiota OTUs compositions in the **(A)** jejunum and **(B)** ileum. The microbiota diversity estimated by **(C–F)** Chao1, Shannon, Simpson, and Observed*_*species in the jejunum and **(G–J)** ileum, respectively. Partial least squares discrimination analysis (PLS-DA) of **(K)** jejunal and **(L)** ileal microbiota community of offspring piglets. Data are represented as means ± SEM, *n* = 8 per group. *P < 0.05. C, control group; A, antibiotic group; P, probiotic group; and S, synbiotic group.

Based on the high-throughput sequencing, a total of 9,791,833 raw reads were generated from 32 jejunum samples and 32 ileum samples of offspring piglets. After removing the low-quality sequences, 7,648,982 effective tags were obtained and clustered into OTUs. Based on 97% sequence similarity, the V3–V4 region sequence was annotated as 9,302 bacterial OTUs in the jejunum, among which there were 541 shared bacterial OTUs; while the V3–V4 region sequence was annotated as 9,488 bacterial OTUs in the ileum, among which there were 469 shared bacterial OTUs (Figures 5A,B).

The piglets from the antibiotic group exhibited a lower alpha diversity in both jejunum and ileum as evidenced by the reduced jejunal indexes of Shannon and Simpson indexes, as well as ileal Chao1 and Observed_species indexes (Figures 5C–J). In the jejunum and ileum, PLS-DA showed that the samples in the control, probiotic, and synbiotic groups were separated and clustered into distinct groups (Figures 5K,L).

### The Microbiota Composition in the Jejunum and Ileum of Offspring Piglets

**Figure 6:**
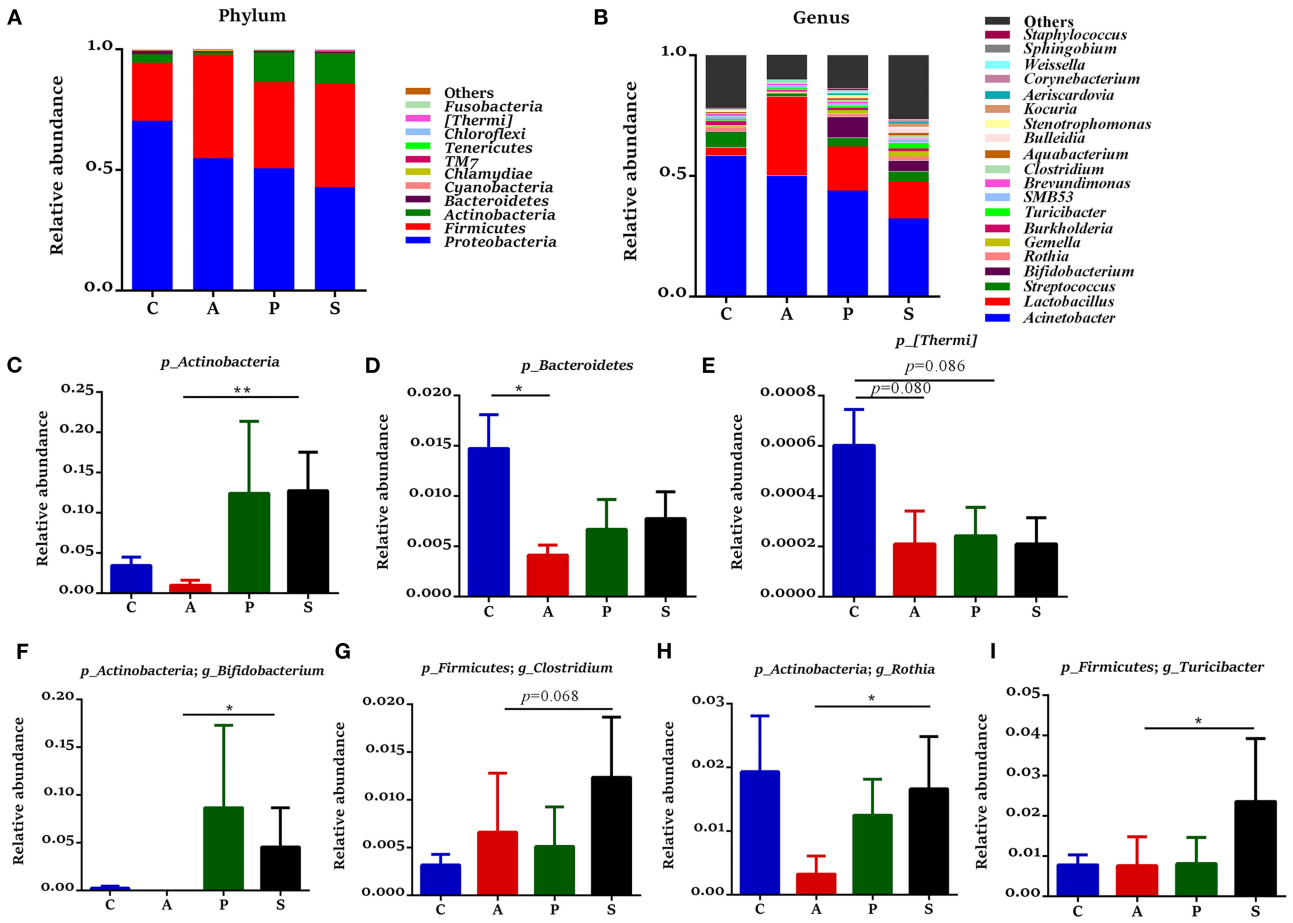
Gut microbiota community composition in the jejunum of offspring piglets. Abundances of the jejunal microbiota at **(A)** phylum and **(B)** genus levels of offspring piglets. All phyla and the top 20 genera were listed. Comparison of relative abundances at **(C–E)** phylum and **(F–I)** genus levels were analyzed by the Kruskal-Wallis rank-sum test. *P < 0.05; **P < 0.01. C, control group; A, antibiotic group; P, probiotic group; and S, synbiotic group.

**Figure 7:**
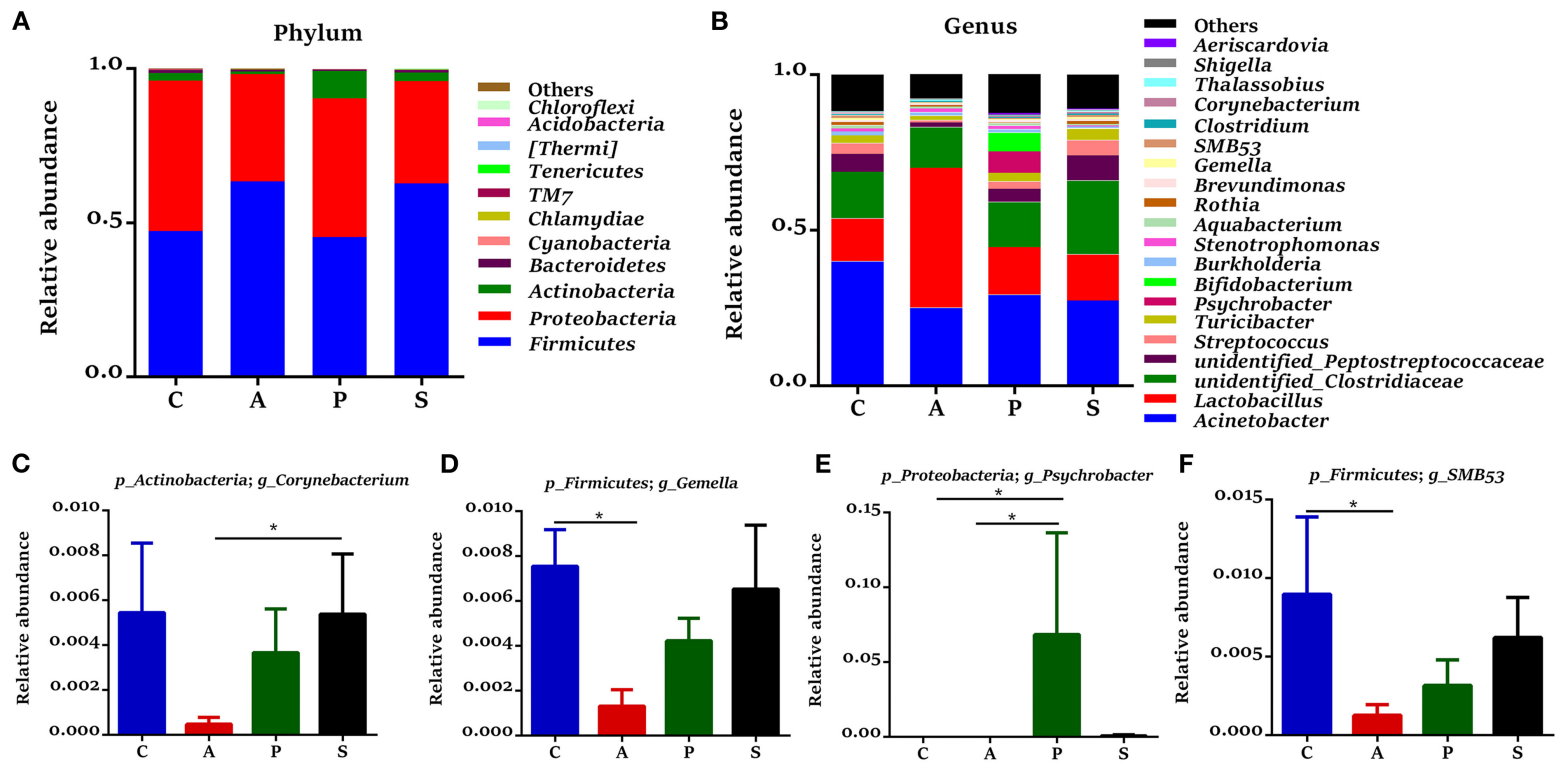
Gut microbiota community composition in the ileum of offspring piglets. Abundances of the ileal microbiota at **(A)** phylum and **(B)** genus levels of offspring piglets. All phyla and the top 20 genera were listed. Comparison of relative abundances at **(C–F)** genus level was analyzed by the Kruskal-Wallis rank-sum test. *P < 0.05. C, control group; A, antibiotic group; P, probiotic group; and S, synbiotic group.

In the jejunum, *Proteobacteria, Firmicutes, Actinobacteria*, and *Bacteroidetes* were the predominant phyla, which accounted for 99.3% of the total bacteria (Figure 6A). The relative abundance of *Actinobacteria* in the synbiotic group was higher (*P* < 0.01) than that in the antibiotic group (Figure 6C), while the relative abundances of *Bacteroidetes* (*P* < 0.05) and *[Thermi]* (*P* = 0.080) were lower in the antibiotic group and as well as the relative abundance of *[Thermi]* (*P* = 0.086) in the probiotic group (Figures 6D,E) when compared with the control group. In the ileum, *Firmicutes, Proteobacteria, Actinobacteria*, and *Bacteroidetes* were the predominant phyla, which accounted for 99.7% of the total bacteria (Figure 7A). However, there was no significant difference at the phylum level among the four groups.

The 20 most dominant microbial genera in the jejunum and ileum are shown in Figures 6B, 7B. In the jejunum, the relative abundances of *Bifidobacterium, Rothia*, and *Turicibacter* were higher (*P* < 0.05) in the synbiotic group, as well as *Clostridium* (*P* = 0.068), when compared with the antibiotic group (Figures 6F–I). In the ileum, dietary probiotic supplementation increased (*P* < 0.05) the relative abundance of *Psychrobacter* compared with the antibiotic and control groups (Figure 7E). In addition, dietary synbiotic supplementation increased (*P* < 0.05) the relative abundance of *Corynebacterium* compared with the antibiotic group (Figure 7C). However, the antibiotic supplementation decreased (*P* < 0.05) the relative abundances of *Gemella* and *SMB*53 compared with the control group (Figures 7D,F).

**Figure 8:**
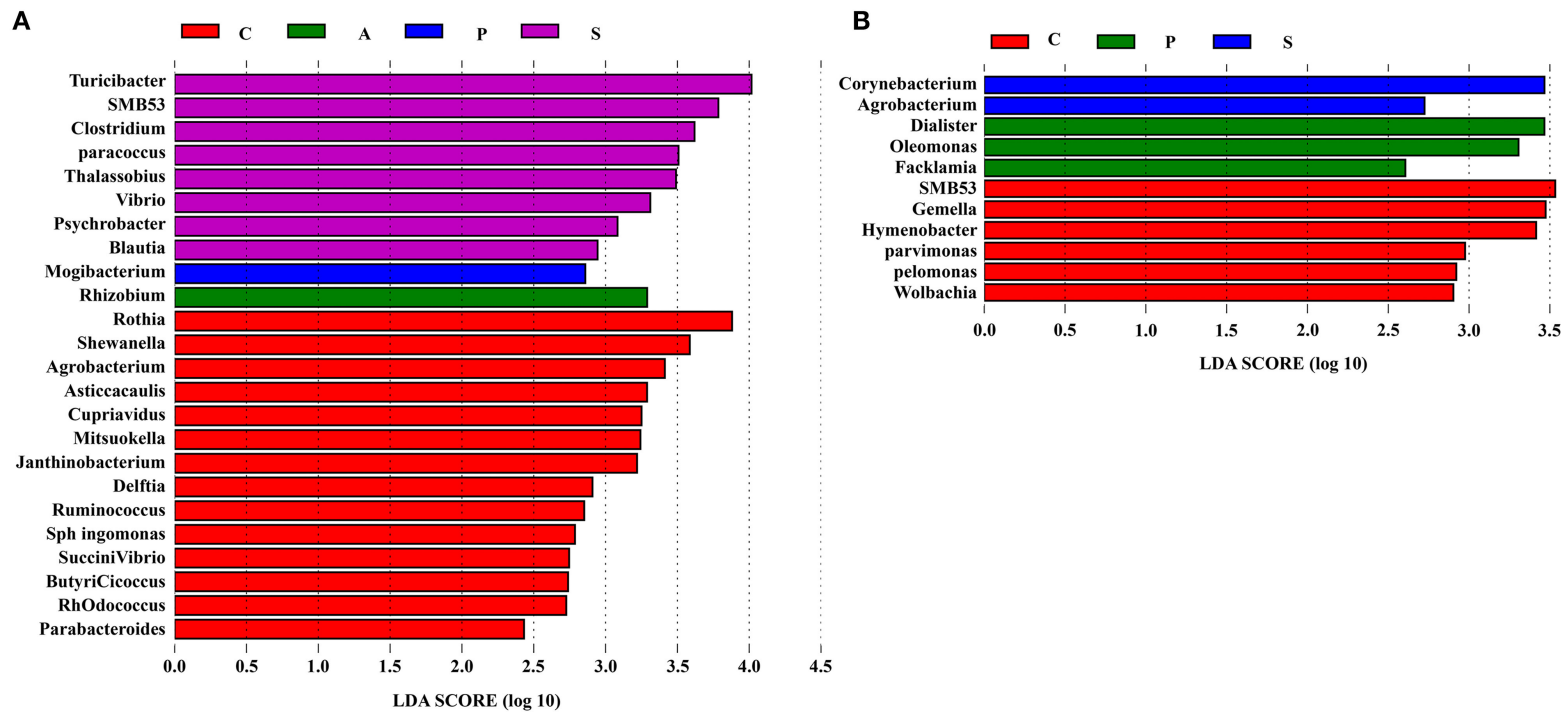
The differentially abundant taxa among the four groups by LEfSe (LDA Effect Size) analysis. Histograms of a linear discriminant analysis (LDA) score (threshold: ≥ 2) in **(A)** jejunum and **(B)** ileum. *n* = 8 per group. C, control group; A, antibiotic group; P, probiotic group; and S, synbiotic group.

To further explore the differences of intestinal microbiota communities among the four groups, the LEfSe analysis was performed at the genus level and presented the LDA score above 2.0. In the jejunum, a significant enrichment of *Turicibacter, SMB*53, *Clostridium, Paracoccus, Thalassobius, Vibrio, Psychrobacter*, and *Blautia* was observed in the synbiotic group, and *Mogibacterium* was the most abundant genera in the probiotic group (Figure 8A). In the ileum, *Corynebacterium* and *Agrobacterium* were enriched in the synbiotic group, while *Dialister, Oleomonas*, and *Facklamia* were enriched in the probiotic group (Figure 8B).

### The Correlation of Intestinal Microbiota, Plasma Parameters, and Intestinal Immune-Related Indexes

**Figure 9:**
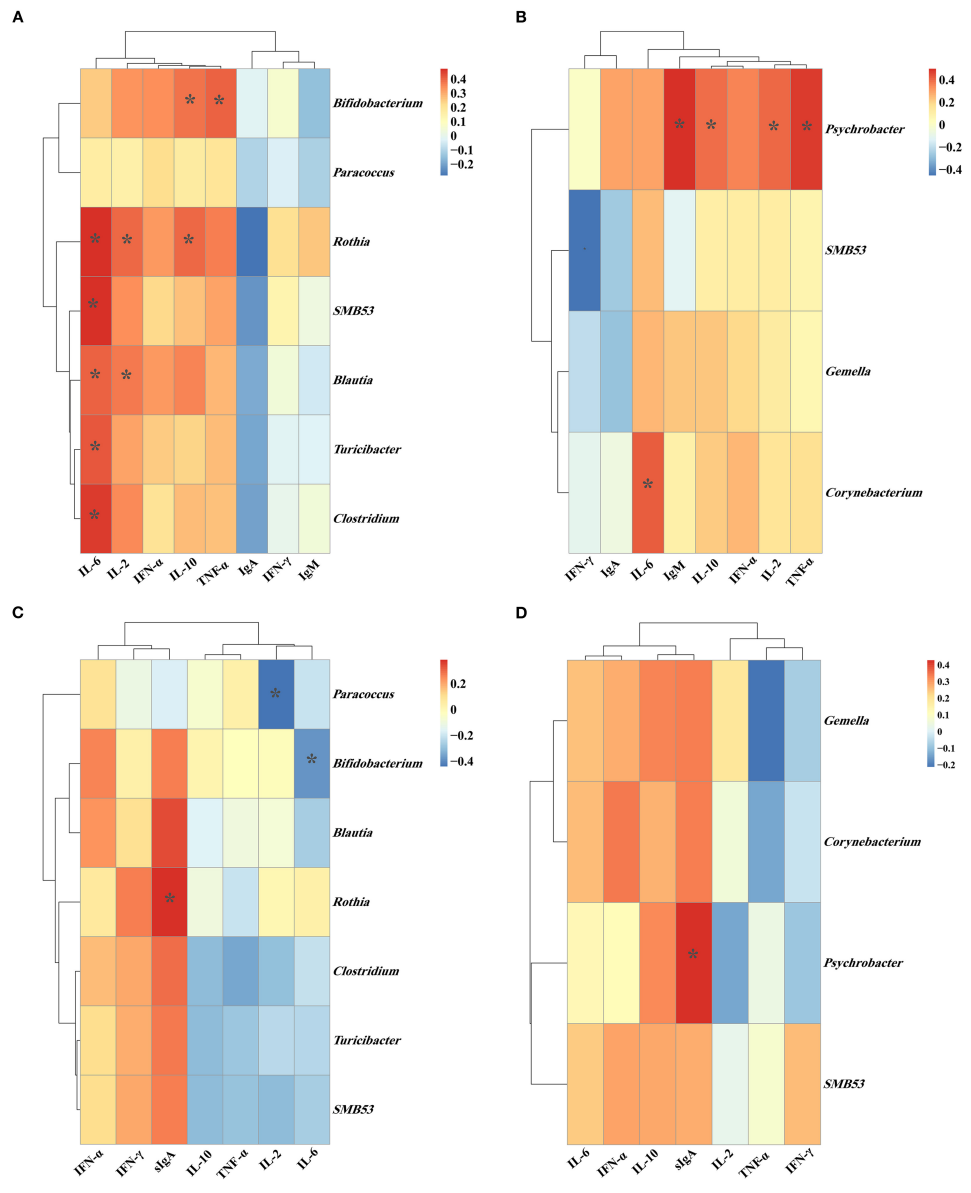
The Spearman correlation between the **(A)** plasma immune-related parameters and jejunal microbiota; **(B)** plasma immune-related parameters and ileal microbiota; **(C)** jejunal immune-related parameters and jejunal microbiota; **(D)** ileal immune-related parameters and ileal microbiota. *P < 0.05. Cells are colored based upon the Spearman correlation coefficient between the significantly altered genera and immune-related parameters; the red and blue represents a significantly positive correlation and negative correlation, respectively.

As shown in Figure 9A, the relative abundance of jejunal *Bifidobacterium* showed a positive (*P* < 0.05) correlation with the plasma concentrations of IL-10 and TNF-α. The relative abundance of jejunal *Rothia* was positively (*P* < 0.05) correlated with the plasma concentrations of IL-2, IL-6, and IL-10. Meanwhile, the relative abundance of jejunal *SMB*53 was positively (*P* < 0.05) correlated with the plasma IL-6 concentration, and the relative abundance of jejunal *Blautia* was positively (*P* < 0.05) correlated with the plasma concentrations of IL-2 and IL-6. Moreover, the relative abundances of jejunal *Turicibacter* and *Clostridium* were positively (*P* < 0.05) correlated with the plasma IL-6 concentration. As shown in Figure 9B, the relative abundance of ileal *Psychrobacter* was positively (*P* < 0.05) correlated with the plasma concentrations of IL-2, IL-10, TNF-α, and IgM.

As shown in Figure 9C, the relative abundance of jejunal *Rothia* was positively (*P* < 0.05) correlated with the jejunal sIgA concentration. The relative abundance of jejunal *Bifidobacterium* was negatively (*P* < 0.05) correlated with the jejunal IL-6 concentration. The relative abundance of jejunal *Paracoccus* was negatively (*P* < 0.05) correlated with the jejunal IL-2 concentration. As shown in Figure 9D, the relative abundance of ileal *Psychrobacter* was positively (*P* < 0.05) correlated with the ileal sIgA concentration.

## Discussion

The gut microbiota of humans or animals is vital for immune system development, metabolism, and mucosal barrier function during early life (1, 2). In human studies, the modulation of microbiota composition by using probiotic or synbiotic during perinatal and early postnatal periods facilitates the colonization of potentially beneficial bacteria in the neonatal gut, which has been considered as a potential dietary strategy to reduce the risk of neonatal disease (29). However, the effects of maternal probiotic or synbiotic supplementation on intestinal health, especially on the immune function of offspring piglets are poorly understood. The present study showed that maternal probiotic or synbiotic supplementation improved intestinal epithelial morphology and enhanced systemic and intestinal immunity to some extent in the subsequent offspring, which is concomitant with alterations of intestinal microbiota composition.

The IgA is a major plasma immunoglobulin that protects the host against microbes. Several studies have reported that *Bifidobacterium bifidum* and *Bacillus subtilis* could increase the levels of immunoglobulins in the host's blood (30, 31). In the present study, maternal dietary probiotic or synbiotic supplementation increased the plasma IgA concentration of offspring piglets, suggesting that these animals gained an enhanced humoral immunity. Thus, our findings further demonstrated the beneficial effect of maternal probiotic or synbiotic supplementation on the systemic immune of offspring piglets.

Intestinal mucosal barrier function is vital to maintain intestinal homeostasis and morphology (32). Increasing studies have demonstrated that several probiotics facilitated the intestinal epithelial barrier function in mammals (30, 33, 34). In the present study, dietary probiotic or synbiotic supplementation to sows improved the intestinal physical barrier function, as evidenced by the increased VH and VH/CD ratio in the jejunum of offspring piglets. Moreover, the reduced intestinal epithelial permeability indicating a favorable mucosa structure for nutrient absorption and digestion (35). These alterations might result in the increased growth performance of offspring piglets.

The immunological barrier function is one of the vital functions for intestinal homeostasis (36). Previous studies have reported that probiotics could induce the macrophages and dendritic cells to secrete immune cytokines to trigger the intestinal immune system (37). *Lactobacillus frumenti* and *Bifidobacterium bifidum* were found to facilitate intestinal sIgA secretion (30, 34). Moreover, *Lactobacillus fermentum* supplementation in rats during pregnancy and lactation periods increased the intestinal IgA concentration in those pups (38). In the present study, maternal probiotic or synbiotic supplementation increased jejunal IFN-α concentration, as well as the jejunal and ileal sIgA concentration in the offspring piglets, which were consistent with previous studies. Recently, *Psychrobacter* bacteria have been recognized as a potential probiotic, which could improve the growth performance and upregulate the immune-related genes (39, 40). In the present study, the increased ileal sIgA might be resulted from the increased abundance of ileal *Psychrobacter*, which were positively correlated with the ileal sIgA. These findings suggested that maternal probiotic or synbiotic supplementation could enhance the intestinal mucosal immunity.

The gut microbial diversity plays an important role in maintaining a stable microbial community (41). In the present study, maternal probiotic or synbiotic supplementation had no effect on the intestine microbial richness and diversity. A recent study also reported that maternal administration of *Lactobacillus fermentum* CECT5716 failed to modify the cecal alpha diversity in offspring rats (38). However, maternal antibiotic supplementation dramatically decreased the microbial alpha diversity in both jejunum and ileum, which is consistent with a previous report that dietary antibiotic supplementation induced microbial dysbiosis by reducing the abundance and diversity of commensal microbes (42). Accordingly, the beta-diversity analysis showed that maternal probiotic or synbiotic supplementation significantly altered the microbial community in the small intestine of offspring piglets.

In the present study, maternal synbiotic supplementation increased the relative abundances of *Actinobacteria, Bifidobacterium, Turicibacter*, and *Clostridium* in the jejunum of offspring piglets. Several studies have reported that *Turicibacter* induces intestinal T-cell activation in the rat hypertension model (43). *Clostridium* (including *Clostridium cluster* IV and XIVa), as a potential probiotic, exerts lots of benefits on intestinal homeostasis (44). *Actinobacteria* (i.e., *Bifidobacterium*) is the key players in maintaining gut barrier homeostasis (30, 45). A previous study also reported that *Bifidobacterium* species can produce acetate and lactate during carbohydrate fermentation (46). The increased abundance of *Bifidobacterium* maybe contributes to the numerically increased ileal acetate concentration in the present study. Acetate, as a major SCFAs, was regarded as the mediator in the crosstalk between the intestinal microbiota and immune regulation (47). These findings may explain the enhanced intestinal mucosal immunity after dietary supplementing synbiotic to sows in the present study. In addition, the jejunal *Bifidobacterium* abundance showed a positive correlation with the plasma TNF-α concentration, and jejunal *Turicibacter* and *Clostridium* abundances were positively correlated with the plasma IL-6 concentration. Therefore, the alteration of these gut microbiota by maternal probiotic or synbiotic supplementation may attribute to the changes of immune-related parameters in the offspring piglets. Further studies are needed to determine the possible causal relationship.

## Conclusion

Maternal probiotic or synbiotic supplementation improved intestinal epithelial morphology and intestinal immunity, and these two additives exhibited similar effects. Alterations in the relative abundances of *Bifidobacterium, Clostridium, Turicibacter*, and *Psychrobacter* in the jejunum and ileum may be associated with intestinal immunity. These findings provide new insight that maternal interventions with probiotic or synbiotic are promising strategies for improving the immune response of offspring piglets by altering the gut microbiota.

## Data Availability Statement

The datasets presented in this study can be found in online repositories. The names of the repository/repositories and accession number(s) can be found at: https://www.ncbi.nlm.nih.gov/sra/?term=PRJNA718786.

## Ethics Statement

The animal study was reviewed and approved by Animal Care and Use Committee of the Institute of Subtropical Agriculture, Chinese Academy of Sciences.

## Author Contributions

XK and KW designed the experiment and wrote the manuscript. CH, WT, MA, QZ, and QH carried out the animal experiment, sample collection, and sample analysis. KW performed the statistical analyses. All authors read and approved the final manuscript.

## Conflict of Interest

The authors declare that the research was conducted in the absence of any commercial or financial relationships that could be construed as a potential conflict of interest.
