# Supplementary material for: The Enhancement of Intestinal Immunity in Offspring Piglets by Maternal Probiotic or Synbiotic Supplementation Is Associated With the Alteration of Gut Microbiota
Source: Front Nutr. 2021 Jul 9;8:686053. doi: 10.3389/fnut.2021.686053 (PMC8298840; doi:10.3389/fnut.2021.686053)
Supplement: Supplementary file 1 [file Data_Sheet_1.pdf]

Supplementary Material

Table S1: Composition and nutrient levels of the sows' basal diets (air-dry basis; %)

Items	Pregnant sows' diet	Lactating sows' diet
Ingredients		
Corn	37.50	66.00
Soybean meal	9.50	25.00
Wheat bran	14.00	5.00
Barley	25.00	
Soybean hull	10.00	
Pregnant sows' premix[1]	4.00	
Lactating sows' premix[2]		4.00
Total	100.00	100.00
Nutrient levels[3]		
Digestible energy (MJ/Kg)	12.55	13.87
Crude protein	13.18	16.61
Crude fiber	4.64	2.80
SID Lys	0.48	0.75
SID Met+ Cys	0.43	0.51
SID Thr	0.37	0.53
SID Trp	0.13	0.17
Calcium	0.59	0.63
Phosphorus	0.43	0.51

Note: [1]Pregnant sows' premix provided the following per kg of diets: $CaHPO_4 \cdot 2H_2O$ 10 g, NaCl 4 g, $CuSO_4 \cdot 5H_2O$ 80 mg, $FeSO_4 \cdot H_2O$ 360 mg, $ZnSO_4 \cdot H_2O$ 240 mg, $MnSO_4 \cdot H_2O$ 100 mg, $MgSO_4 \cdot 7H_2O$ 1 g, 1% ICl 50 mg, 1% Na_2SeO_3 36 mg, 1% $CoCl_2$ 16 mg, $NaHCO_3$ 1.4 g, VA 10 000 IU, VD_3 1 800 IU, VE 20 mg, VK_3 2.4 mg, VB_1 1.6 mg, VB_2 6 mg, VB_6 1.6 mg, VB_{12} 0.024 mg, folic acid 1.2 mg, nicotinamide 20 mg, pantothenic acid 12 mg, biotin 0.12 mg, ferrous glycinate 100 mg, choline chloride 1g, phytase 200 mg, fruity 80 mg, limestone 12 g.

[2]Lactating sows' premix provided the following per kg of the diet: $CaHPO_4 \cdot 2H_2O$ 10 g, NaCl 4 g, $CuSO_4 \cdot 5H_2O$ 80 mg, $FeSO_4 \cdot H_2O$ 360 mg, $ZnSO_4 \cdot H_2O$ 240 mg, $MnSO_4 \cdot H_2O$ 100 mg, 1% ICl 50 mg, 1% Na_2SeO_3 36 mg, 1% $CoCl_2$ 16 mg, $NaHCO_3$ 1.4 g, VA 10 000 IU, VD_3 1 800 IU, VE 20 mg, VK_3 2.4 mg, VB_1 1.6 mg, VB_2 6 mg, VB_6 1.6 mg, VB_{12} 0.024 mg, folic acid 1.2 mg,

nicotinamide 20 mg, pantothenic acid 12 mg, biotin 0.12 mg, Lysine 1.5 g, ferrous glycinate 100 mg, choline chloride 1g, phytase 200 mg, fruity 80 mg, limestone 12 g.

[3] Energy and amino acids is a calculated value, while the others are measured values. Calculated nutrient levels using values for feed ingredients from the NRC (2012). SID, standard ileum digestible.

Table S2: Composition and nutrient levels of the basal diet for weaned piglets

Items	Prophase nursery diet (35-65 days of age)
Ingredients (%)	
Corn	54.92
Soybean meal	22.00
Wheat bran	10.13
Rice bran	8.95
Premix[1)]	4.00
Total	100.00
Nutrient levels[2)]	
DE/(MJ/kg)	13.50
CP	15.65
Ca	0.46
TP	0.51
Thr	0.79
Ser	0.72
Met	0.31
Ile	0.68
Leu	1.40
Lys	1.36

Note: [1]Premix provided the following per kilogram of diets: enzymic preparation 1.2 g, VA 26 000 IU,VD_3 10 000 IU,VE 70 IU,VK_3 10 mg,VB_1 10 mg,VB_2 25 mg,VB_6 10 mg,VB_{12} 0.075 mg, biotin 0.4 mg, folic acid 5 mg, nicotinamide 100 mg, pantothenic 50 mg, choline 1 600 mg, flavoring agent 500 mg, edulcorant 300 mg, acidulating agent 5 g, Cu (as $CuSO_4 \cdot 5H_2O$) 230 mg, Mn (as $MnSO_4 \cdot H_2O$) 97 mg, Zn (as $ZnSO_4 \cdot H_2O$) 218 mg, Fe (as $FeSO_4 \cdot H_2O$) 165 mg, I (as $Ca(IO_3)_2$) 0.3 mg, Se (as Na_2SeO_3) 0.3 mg, Co (as $CoSO_4 \cdot xH_2O$) 0.4 mg, glucose 2.1 g, antioxidants 0.4 g, antimildew agent 1 g, Ca (as $CaHPO_4$ and $CaCO_3$) 3.42 g, P (as $CaHPO_4$) 1.155 g.

[2] Energy is a calculated value, while the others are measured values.

Table S3: The concentrations of short-chain fatty acids (SCFAs) in jejunum and ileum of piglets (n = 6, μg/g)

Items	Control group	Antibiotic group	Probiotic group	Synbiotic group	SEM	*P*-values
**Jejunum**						
Acetate	137.39	112.80	107.73	123.85	13.16	0.878
Butyrate	38.44	51.66	47.28	39.27	5.06	0.778
**Ileum**						
Acetate	487.96	582.69	501.99	736.08	55.41	0.405
Butyrate	43.89	89.50	63.13	37.94	8.17	0.083

Data are means ± SEM. Differences were considered significant at ($P < 0.05$).
